# Understanding Functional Evolution in Orthologs and Paralogs

**DOI:** 10.1007/s00239-025-10274-4

**Published:** 2025-10-14

**Authors:** Maeva Perez, Katherine Hurm, David A. Liberles

**Affiliations:** 1 Department of Biology, Hong Kong Baptist University, Hong Kong SAR, China; 2 Department of Biology and Center for Computational Genetics and Genomics, Temple University, Philadelphia, PA 19122 USA

**Keywords:** Gene duplication, Orthologs, Paralogs, Molecular biophysics, Biochemistry, Evolutionary theory, Stochastic modeling

## Abstract

The Quest for Orthologs has focused on identifying orthologs from the perspective that they are more likely to have retained function over a given evolutionary distance than paralogs (or xenologs) have, enabling the transfer of functional annotation. It has become clear that function is defined by biochemistry that is under selective pressure. Quantitative descriptions of function are available within this framework and may offer understanding that is not provided by more qualitative descriptions of function. Changes in selected biochemistry, mutational processes, and selective strength can all lead to quantitative changes in function. This is discussed for proteins that have been subjected to gene duplication and for proteins that have evolved simply through the speciation process.

## Introduction

In the era of genome sequencing, protein-encoding genes can be identified by having long open reading frames and evidence of transcription, but most are identified by having sequence similarity with other known genes from other species, suggesting homology (descent from a common ancestor) (Langschied et al. 2024). Once genes have been identified, the next question becomes, what is the function of a particular sequence? Functions are ultimately defined biochemically, in that the properties of a sequence drive the potential biochemical interactions with other molecules in a cell. Biochemical experiments and screening of complete proteomes across the tree of life established that the diversity of biochemical functions we see across proteins today arose from the gradual change and the modular recombination of relatively few protein domains (Chothia et al. 2003; Caetano-Anollés et al. 2012).

Numerous functional annotation systems based on the biochemical properties of proteins and their associated hierarchical classification structures have been developed to represent biologically significant functions (for example, the Enzyme Commission (E.C) classification for enzymes (The UniProt Consortium 2025), the Gene Ontology (Gene Ontology Consortium 2004)). Although these systems have proven valuable in various contexts, they share a common limitation in that the biological interpretations derived from them are constrained by the predefined functional categories they employ. Furthermore, this assignment of biochemical functions to gene products based on their sequences omits the fact that protein functions are also affected by their environmental context. Lastly, the functional description they give is qualitative.

From a biochemical perspective, proteins do three things. They bind to other molecules, bind to other molecules, and catalyze reactions (here in many contexts, this can be thought of as simply binding, but to the reaction transition state), and they transport molecules between compartments. The function is described quantitatively by what they bind to and what they do not bind to (or transport).

**Fig. 1:**
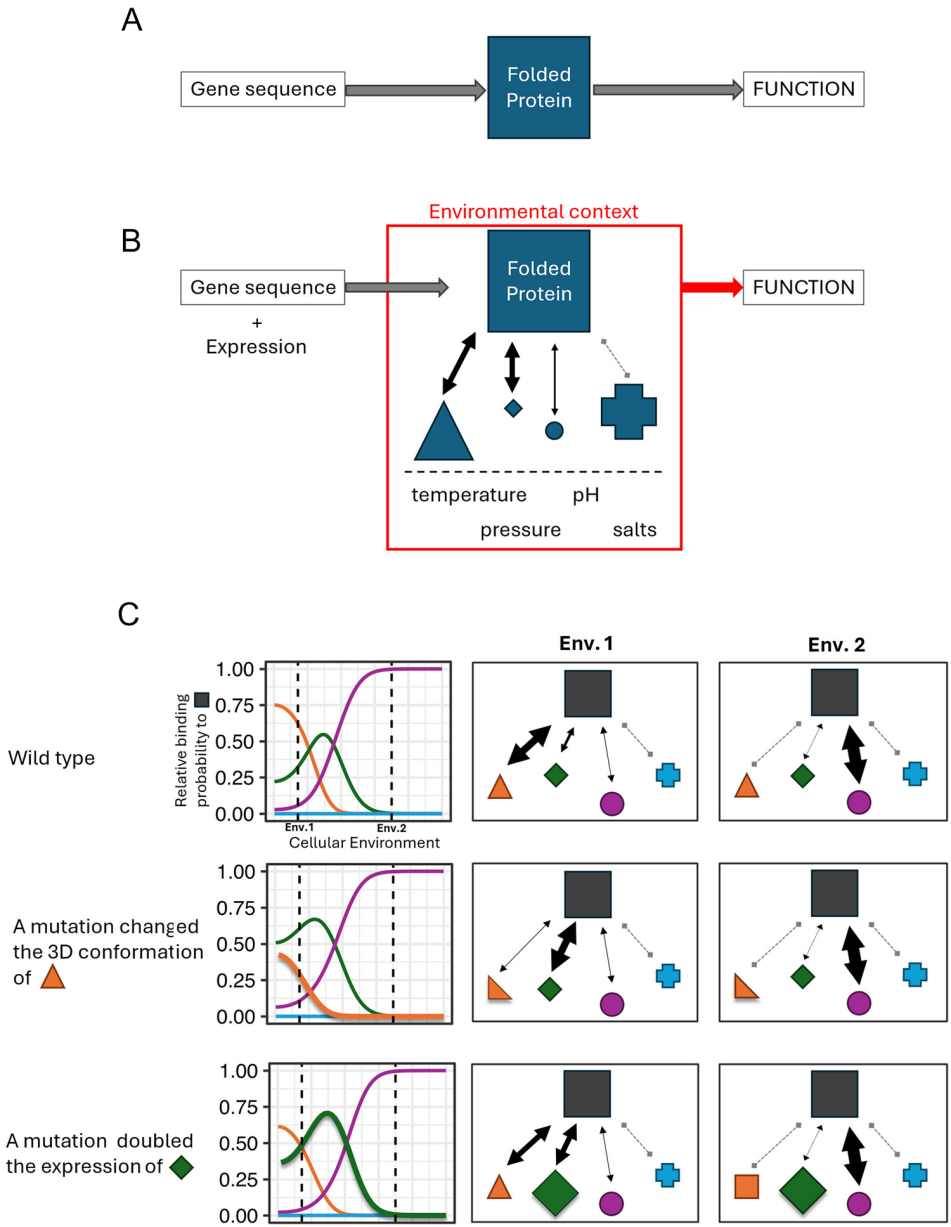
Different definitions of protein functions. **A** The qualitative definition of protein function where the biochemical function of a protein is defined by its 3D conformation which is encoded in the gene sequence. **B** The quantitative definition of protein function where the biochemical function of a protein is dependent upon both its gene sequence but also its expression relative to other proteins defining a network of interactions, and other factors of the cellular environment (e.g., temperature) which together constitute a unique environmental context. **C** The relative probability of interactions of a protein (gray square) to other proteins is defined across environmental parameters by a Boltzmann distribution so that different cellular environments result in different networks of interactions. Mutations that change either the structure (middle panel) or relative concentration (bottom panel) of interacting proteins bring change to the Boltzmann distribution. The bolded lines in the line plots on the left highlight the changed binding potential. In the schematic representations on the right, different shapes represent different proteins. The shape sizes represent their relative concentration. The arrows and their thickness represent the interactions and their strengths in terms of relative binding probabilities. A negative interaction (e.g., not-binding) is also represented as a gray dashed link

The totality of interactions is defined by a Boltzmann distribution to describe the fractional binding occupancy of any molecule about any other molecule in the context of the entire proteome available to interact with (Dill and Bromberg 2011). In other words, the specificity of interaction depends upon the relative concentration of the protein in the cell and that of each of its potential interacting molecules as well as the inherent sequence-dependent binding affinities of the molecules (Fig. 1). At different cellular concentrations, for instance, the *Bacillus licheniformis* protease *(BLP*), an enzyme able to break down a variety of proteins, preferably hydrolyzes different peptide bonds (Butré et al. 2014). Other extreme examples of context-dependent functions are found in so-called “moonlighting” or “gene sharing” proteins (Turek and Irving 2021; Gupta and Uversky 2023). These proteins have totally different functions in different tissues even though they are expressed by the same genes and are identical at the sequence level. The vertebrate lactate dehydrogenase (*LDH*) B4 enzyme is an early example of such a protein (Hendriks et al. 1988). When expressed in the eyes instead of the heart, its catalytic substrate is absent and the protein functions instead as an essential component of the lens crystallin.

For enzymes, the quantitative description of proteins is found in Michaelis–Menten kinetics, where the described equilibrium from the kinetic equation is bound by the energetics according to Haldane’s Relationship (Haldane 1930). Key parameters include *k*_cat_ (the rate of catalysis of a bound substrate molecule) and *k*_*M*_ (the Michaelis constant reflecting the affinity of an enzyme to bind its substrate), as well as inhibitory constants and binding constants associated with allosteric regulation. For transporters, the key summary descriptor would be a rate of transport, although this can be broken down to more mechanistic components, all with their own parametric descriptors.

Since changes to the sequence of either the protein itself or to other proteins encoded in the genome will change the terms of the Boltzmann Distribution, core attributes of selectable functions may be more quantitative than common descriptions of functions would lead us to believe. When function is defined under the light of its environmental context, changes can be readily divided into cis changes that change the protein’s sequence or expression level and trans changes that change the coding sequence or expression level for interacting proteins (or other molecules through downstream effects). Considering both cis- and trans- effects is instrumental for understanding evolution at higher biological scales such as that of whole metabolic pathways and physiology. For instance, studying the long-term evolution of the glycolysis pathway, Orlenko et al. (2016a) found that sequence evolution led to variability in flux control over the pathway, although it was unclear if this was the result of directional selection. In simulation, Orlenko et al. (2016b, 2017) found that changes in the kinetic properties of enzymes from divergence can lead to shifts in points of flux control of the whole pathway which in turn affect the selective pressure and evolution of the enzymes involved in the other steps. This can occur without directional selection on the pathway as a whole but can also be driven by directional selection. Disentangling functional shifts in individual proteins driven by only epistatic effects and those driven by directional selection (and by corresponding epistatic effects) is an open problem. Further, if function is defined quantitatively, not only can it shift due to selection on the biochemical optimum, but it can also shift due to demographic and genetic factors independent of that selection, like changes in the mutation rate that affect mutation-selection-drift equilibrium or changes in the organism’s effective population size that affect the strength of selective factors. Increased mutational acceptance can lead to a change in the ensemble of interacting proteins described by the Boltzmann Distribution, as the energies of each individual interaction can change as the molecules (sequences) change.

Furthermore, even when the environmental context is taken into account, the biochemical definition of what a protein does is not sufficient to define function, as selection is also a necessary ingredient. If a function doesn’t influence fitness or the ability of an individual to survive, mate, and reproduce, that biochemical characterization is ultimately irrelevant. Indeed, a protein must also be under selection to retain a specific function, even if transiently on evolutionary timescales. This point was emphasized through the back and forth between evolutionary researchers and the ENCODE project, which aimed to build a comprehensive map of functional elements in the human genome (Graur et al. 2013; Doolittle 2013; Kellis et al. 2014; Snyder et al. 2020), was emphasized. While de novo proteins would lack evidence for selection on their functions, absence of evidence is not evidence of absence, and some newly arisen proteins may become under selection for their newly emerged functions. If selection on a protein changes between species, this is then relevant to consideration of function in the classification of homologs, including orthologs.

The next sections will discuss the functional evolution of orthologs and paralogs and highlight how new insights are brought about by integrating a quantitative dimension.

## Functional Evolution of Orthologs

The field of genomics centered on the “quest for orthologs” from a view that originates with Ohno suggesting that in the absence of gene duplication, evolution is conservative and that gene duplication through the generation of redundancy leads to protein functional change (Ohno 1970). A correlate of this is that orthologs are unlikely to have changed function; the ortholog conjecture. The view attributed here to Ohno is perhaps a straw man argument, although the practice of functional annotation transfer by reciprocal best BLAST hit is unfortunately consistent with that straw man argument.

It is within biological reason to assume that those genes descended from a common ancestor maintain the same/similar function. Compared to paralogs, which relax selection when in a redundant state, it is expected that these genes functionally diverge more slowly as orthologs. When in a duplicated state, the productive function of the genes only needs to occur in the sum of the two genes rather than in a single gene, enabling the pair of genes to both potentially evolve faster and sample new sequences and functions. It should be noted that genes also can evolve deleterious interactions, and these would not necessarily occur at an appreciably faster rate in orthologs than in retained duplicates. We will also argue that these interactions are not really “functions”.

The ortholog conjecture has been previously challenged and discussed by Nehrt et al. (2011). They tested 8,900 genes of mice and humans in functional assessments and found that paralogs had more functional similarity compared to orthologous genes when both had high sequence identity (Nehrt et al. 2011). These claims were eventually tested once again by Altenhoff et al. (2012). The study focused on analysis from the well-characterized system involving *Saccharomyces cerevisiae* and *Schizosaccharomyces pombe* and a 13-pair species-wide study. The results were consistent with orthologs having more similar GO annotations than paralogs when presented with similar sequence divergence. From this previous characterization, which had argued against the ortholog conjecture, the finding was found to have potential authorship bias and variable frequency of use of GO terms across species (Altenhoff et al. 2012).

So what explains such discrepancy? The discrepancy is due to properly accounting for bias, and the ortholog conjecture is indeed generally supported. Beyond computational analysis, there is experimental support for the accepted evolutionary dynamics of a role for duplication in enabling greater sampling of mutational space (Mihajlovic et al. 2025).

But there are other points to consider that are consistent with the ortholog conjecture. If orthologs are defined by the node of last common ancestry separating any two proteins, they may have fixed duplication events in their history. Mutations can occur in the duplicated state that eventually go to fixation, even when the extra copy does not go to fixation. This process is expected to both be extremely common and impossible to precisely track. For instance, a large body of literature has shown that following whole-genome duplication, most genes eventually return to a single copy state (Van de Peer et al. 2003; Martens and Van de Peer 2010; Konrad et al. 2011; Lien et al. 2016), thereby creating cases of hidden paralogy. Adding to this complexity, genes are most often considered as the unit of orthology despite the fact that they are themselves modular entities and that the true unit of homologies is at the levels of their functional domains (Gabaldón and Koonin 2013). Thus, a super-ortholog, as defined by Zmasek and Eddy (2002), is a gene that shows no evidence for gene duplication and/or domain-architecture recombination in its history; it probably does not exist in its pure sense. Secondly, even with negative selection to retain function, sequence changes do accumulate over time (Illergård et al. 2009). So, a core question becomes: what is the rate at which orthologs diverge in function, and what are the factors that determine that rate?

Answering this question is difficult because while it is classically stated that sequence begets structure begets function (Illergård et al. 2009), there is noise in the links at each level. On the one hand, the chemical properties of amino acids are somewhat redundant, and the structure of proteins is tolerant to small sequence/biochemical variation. As a consequence, only a subset of changes that accumulate as sequence divergence causes structural divergence; thus, the divergence between closely related proteins can be an order of magnitude higher at the level of the sequences than at the level of their 3D structure (Illergård et al. 2009). On the other hand, epistatic genetic interactions within and across proteins cause additional discrepancies between the observed sequence structure and qualitative functional divergence. For example, several combinations of mutations of the interacting Fos and Jun proteins can maintain the proper properties of the heterodimer and thus its specific identity as the transcription factor AP-1 (Ransone et al. 1989; Diss and Lehner 2018).

Epistatic interactions are thought to be a key factor in the functional evolution of orthologs because they go beyond predictable protein–protein interactions to include complex networks of interacting proteins. By opening up a multitude of new paths in the mutational landscape toward the same functional outcome, epistasis is expected to decrease the rate of functional divergence across orthologs on evolutionary time scales (Starr and Thornton 2016).

To evaluate this expectation, we must consider not only the networks of interacting proteins but also the dynamics of these networks within biological pathways. The context dependence of quantitative functional evolution of enzymes dependent upon other activities in the pathway was studied in simulation under stabilizing, directional, and periodic selection regimes (Orlenko et al. 2016b, 2017). These studies revealed that even in populations at mutation-selection-drift equilibrium, stabilizing selection acting at the level of the metabolic pathway will, over time, lead to shifts in the flux-controlling steps because the enzymes within the pathway are evolving co-dynamically. In contrast, greater evolutionary stability of the flux-limiting steps in a metabolic pathway can be achieved under specific dynamic demographic scenarios underlying the co-evolution between functional activity and expression level. Taken together, these observations highlight that epistasis can also promote the functional divergence of orthologous genes, an observation further supported by the fact that the evolutionary rates of proteins epistatically associated are correlated (Schlosser and Wagner 2008). The time scales and protein localization of this correlation can remain complex.

## Functional Evolution of Paralogs

**Fig. 2:**
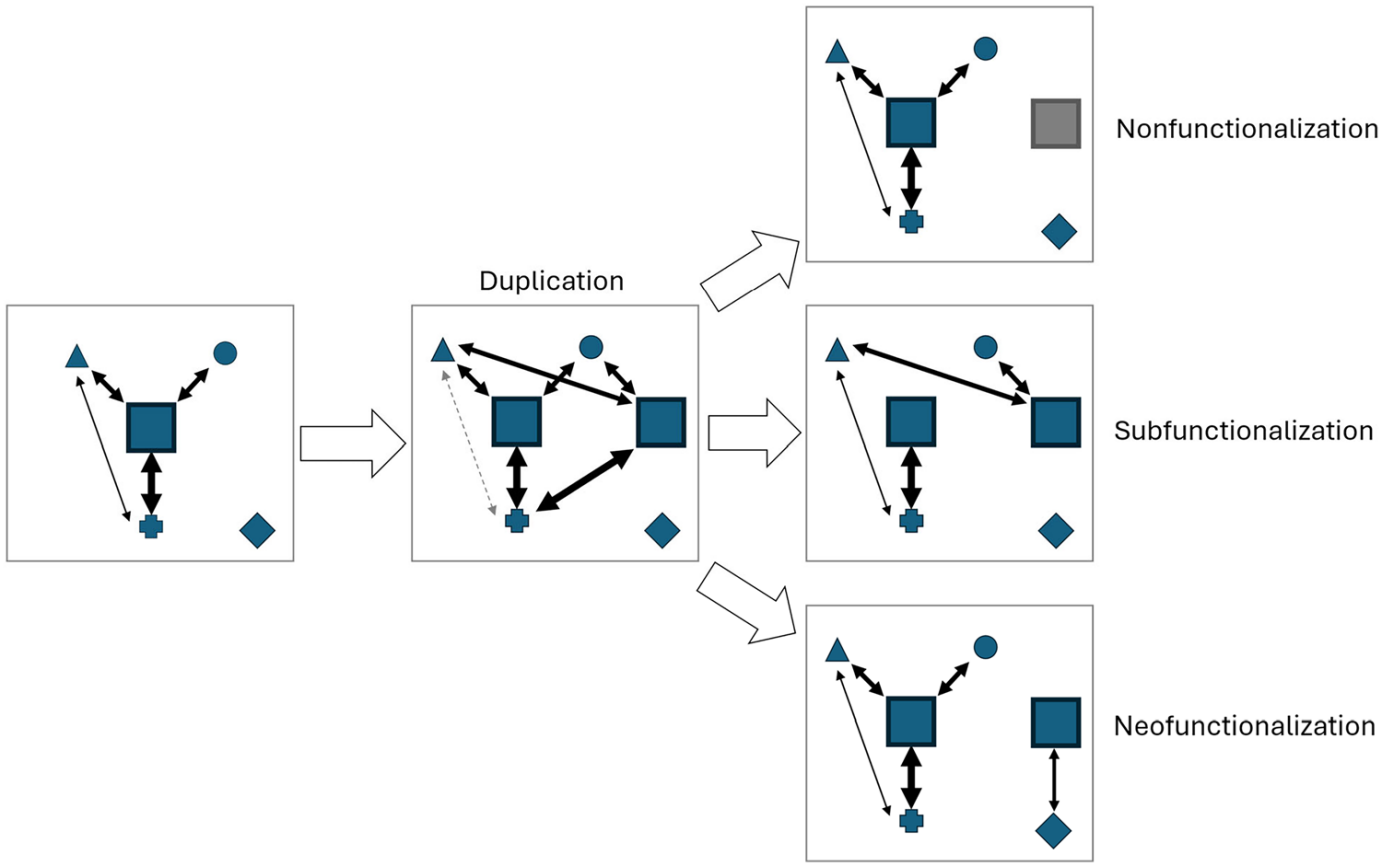
Fate of paralogs following duplication. A protein function is defined by the network of interactions it forms with other proteins in its environment. Following gene duplication, the dosage balance of that protein is changed, and the resulting stoichiometric shift will disturb that network. This disturbance is represented as a loss of interaction (dashed gray arrow) between two proteins due to competition with the interacting paralogous copies. The network is re-established following nonfunctionalization, subfunctionalization or neofunctionalization. Blue shapes represent proteins. The arrows and their thickness represent the interactions and their strengths (i.e., binding probabilities)

Gene duplication plays a crucial role in the evolution of novel gene functions. This process can occur at various scales, ranging from the duplication of individual genes (or even domains within genes) to entire genome duplications through diverse mechanisms. Following a duplication event, the resulting paralogous genes face two possible fates: the loss of the extra copy through pseudogenization or its retention with modifications. These modifications lead to either subfunctionalization or neofunctionalization (Fig. 2). Subfunctionalization occurs when the paralogs evolve complementary functions, effectively partitioning the ancestral gene’s role. This partitioning can manifest as (1) temporal specialization, such as in the fetal and adult hemoglobin (Groudine et al. 1983); (2) tissue-specific expression, as exemplified by Engrailed 1 and 2 in zebrafish (Higashijima et al. 2004); or (3) structural/modular specialization, such as multimerization or the separation of catalytic and substrate-binding activities in certain enzymes (Tocchini-Valentini et al. 2005; Pereira-Leal et al. 2007). In contrast, neofunctionalization involves the acquisition of an entirely new function by one of the paralogs, independent of the original function. A striking example is the evolution of opsins in deep-sea fish, where gene duplication followed by spectral tuning in the resulting copies has enabled adaptation to different light environments (Musilova et al. 2019). The new function that emerges can but needn’t correspond to the loss of the ancestral function. In some cases, a neofunctionalized protein will still perform its ancestral function in addition to the new one.

The fates of duplicated genes, including pseudogenization, neofunctionalization, and subfunctionalization, are shaped by a complex interplay of neutral and selective processes. Neutral processes are primarily responsible for the loss of duplicate genes through the accumulation of deleterious mutations and genetic drift leading to pseudogenization. This neutral loss process can take a long time to play out under several different mechanisms (Johri et al. 2022; Wilson and Liberles 2023b). While gene loss convergently following whole-genome duplication events across the tree of life suggests selective forces may also play a significant role (Albalat and Cañestro 2016), in consecutive whole-genome duplication events, the same genes were not consistently retained or lost (Lien et al. 2016; Hermansen et al. 2016; Wilson and Liberles 2023b). In flowering plants, for instance, 50% of core genes have rapidly returned to single copy following whole-genome duplication events (Li et al. 2016), but there is a bias toward loss for genes with housekeeping functions and genes with high and ubiquitous expression. These observations could be explained by a selective pressure to maintain dosage balance (Shi et al. 2024), or by a selective advantage to losing a redundant gene copy due to the inherent fitness cost of protein production and expression (Wagner 2005); a hypothesis referred to as the "black queen hypothesis" (Mas et al. 2016) or "regressive evolution" (Albalat and Cañestro 2016). There may also be other selective pressures acting, including against the duplication of genes that have the propensity to evolve spurious deleterious interactions (Liberles et al. 2011). Along these lines, Doolittle et al. (2014) have suggested the importance of differentiating between selected biological functions and biochemical effects that may be selectively neutral.

The informational-operational hypothesis that affects which genes are laterally transferred may also be at work with gene duplication (Jain et al. 1999). Along these lines, there will be a mutational bias to lose genes that simply are not beneficial to keep in a duplicated state and that may affect many informational genes.

Subfunctionalization can also be explained by neutral and selective models. The complementary degeneration of domains (CDD) model (Force et al. 1999) suggests neutral processes lead to each paralog losing a different subset of the ancestral gene’s functions. However, there is complexity not described in that model. Wilson and Liberles (2023b) showed that dosage balance can present a selective barrier to regulatory subfunctionalization. Further, subfunctionalization of binding interactions in the coding sequence involves the gain of specificity, and the reduction in sequence space associated with a more specific protein may require selection (Liberles et al. 2011).

There may also be a selective pressure for paralogs to subfunctionalize when the duplicated genes are in competition or when the ancestral gene performed multiple functions that were in conflict (i.e., the escape from adaptive conflict (EAC) model). Unlike nonfunctionalization and subfunctionalization, neofunctionalization is thought to result only from selective processes (although this may not be true for binding functions that relax affinity-specificity tradeoffs (Liberles et al. 2011)). The classical model of neofunctionalization states that thanks to functional redundancy generated by gene duplication, purifying selection to retain the original function is relaxed in one of the gene copies, enabling the accumulation of mutations which can eventually confer a new activity (Ohno 1970). Another model coined ‘innovation–amplification–divergence’ (Francino 2005) reflects the idea formulated by Baldwin (Baldwin 1896) that this adaptive evolution is facilitated by phenotypic plasticity. The model proposes that the new fitness-improving function appears before duplication (e.g., the capacity for an enzyme to bind a new substrate) but is not used because the genetic/cellular context favors the ancestral function. It is after duplication, thanks to relaxed selection for the maintenance of the original function in one of the copies, that the new function can be expressed preferentially. The fitness gained from the expression of this new function may in turn relax the selective pressure on the initial function/other gene copy, leading to its loss. In this model, neofunctionalization thus evolves from subfunctionalization. Modeling an evolving population of genes that form protein lattices and bind a ligand, Rastogi and Liberles (Rastogi and Liberles 2005) showed that neofunctionalization was more likely to arise from subfunctionalization than de novo. However, it has also been shown that there is an enzyme-specific capability to what evolution is possible (Schmutzer et al. 2024).

With an expectation that duplicate genes can change function (as can orthologs), models enable quantification of these rates. While change in GO term could be modeled over a phylogeny as a basic Poisson process, we have argued in this piece why the utility of this approach may be limited. The assumptions of this process include a constant time-dependent rate of change of function independent of other biochemical attributes of sequence or their evolutionary potential. A discussion of different classes of models follows below. A recent review of some modeling frameworks has been presented (Assis et al. 2024).

## Mechanistic Models Bring New Insights into the Dynamics of Sub-neo- and Non-functionalization

Many phenomenological models aim to explain how genes may gradually change in function, but they are limited in that they cannot make quantitative predictions about the rate or dynamics of these evolutionary changes. What is the dynamic and interplay between the neutral and selective processes at play following gene duplication? How and under which circumstances does dosage balance affect the fate of paralogs? How do genes survive following horizontal gene transfer? By experimentally evolving populations in silico under different assumptions of biological parameters and processes, modeling approaches enable us to fill some of these important knowledge gaps. Some of the aforementioned questions have been tackled in preliminary ways using mechanistic models (for example, with the role of dosage balance), while others (for example, involving lateral gene transfer) are much less well explored (see Assis et al. 2024 for a review of some of these models).

From small scale duplications (SSD) affecting single genes or domains within, to whole-genome duplications (WGD), the duplication of genetic material can happen at a wide range of scales. One key differentiating factor between SSD and WGD is whether genes are duplicated together with their interacting partners. This distinction is significant because genes that are sensitive to dosage constraints may follow different evolutionary paths based upon whether they are duplicated together with binding partners or not. In plants, organellar and some informational genes show a bias toward returning to a single copy in multiple lineages after whole-genome duplication (Li et al. 2016). Alternatively, many metabolic genes are found in multicopy following whole-genome duplication events.

Other models include those on duplicate gene retention after consecutive WGD events using the gene duplicability hypothesis. This hypothesis states that the number of interacting partners of a gene can be used to explain the loss or retention of it. Certain genes can benefit from consecutive duplication events, as seen in the multiple copies of *AMY1* in *Homo sapiens* compared to our primate ancestors. Other genes with dosage sensitivity and more functional constraints, like cell cycle regulators *TP53*, do not generally benefit from extra copies. The biochemistry of the underlying duplicates is not clear. In the case of *TP53* in elephants, there is an ongoing debate about any selective advantage (or not) for these duplicates (Callaway 2015; Nunney 2022; Joerger et al. 2025). Modeling can explain these gene retention observations as well as other paralogs which arise from WGDs such as *HOX* genes and many in polyploid species (Singh and Krumlauf 2022; Deem and Brisson 2024). Wilson and Liberles (2023a) have established the time and function-dependent complexity of the gene duplicability hypothesis. This modeling framework currently exists for the evaluation of genome-wide collections of genes, but has not yet been extended to evaluate expectations for individual genes.

Early modeling of dN/dS for gene duplicates shows that following duplication, dN/dS decays exponentially to an asymptote from a higher instantaneous intercept at the point of duplication (Hughes and Liberles 2007). So, with enough time, duplicates begin to behave like non-duplicated genes. But duplicate genes that are retained in genomes are retained with modifications. The link between changing selective constraint over time and retention over time has not yet been built into a mechanistic model.

This faster sequence evolution is often viewed in early duplicates. At the early stages of duplication, genes exhibit this reduction of modular function, which opens up the doors for functional divergence. Redundancy in early paralogs due to shared functions allows for high rates of mutations, thus suggesting lower rates of purifying selection. What happens to these module duplicates can be categorized into the previously mentioned subfunctionalization, neofunctionalization, or specialization events. Often, these duplicates retain the same or similar functions, and the specialization of the duplicate gene diversifies or refines the ancestral function.

The subsequent events occurring after gene duplication ultimately lead to the duplicates’ fate. Understanding the underlying biochemical mechanisms acting on the duplicate allows for a better idea of duplicate trajectory and evolution. In recent decades, models have been constructed to explain duplicate retention or loss based on neofunctionalization, subfunctionalization, and dosage balance events. Konrad et al. characterized a model with expectations for time-dependent retention associated with these different fates and the underlying processes generating them. Subfunctionalization was the dominant strategy when applied to *Oikopleura dioica* duplicate data (Konrad et al. 2011).

Using a different modeling framework that integrates biophysical and population genetics parameters, Wilson and Liberles (2023a) characterized the rate of subfunctionalization for genes under dosage balance constraints under SSD or WGD scenarios. The authors showed that the same selective pressure to maintain dosage balance resulted in dramatically different trends of gene retention over time. Following SSD, selection against dosage imbalance led to the rapid culling of duplicates through non- and subfunctionalization. In contrast, the stoichiometric balance of proteins tends to be better maintained following WGD and, consequently, selection acting against dosage imbalance tends to reduce the rate of stoichiometry-breaking sub- and non-functionalization, leading over time to more gene pairs to be retained in the genome. This selective pressure also indicates that subfunctionalization does not result purely from neutral processes as previously described. Interestingly, the type of Markov modeling framework the authors used in this study can be extended straightforwardly to neofunctionalization and to the evolution of genes without duplicates.

Subfunctionalization reduces the number of modular functions and the future potential for retention through duplication as well as the expected segregation time for duplication events that lead to an elevated substitution rate during periods of redundancy (Stark et al. 2017). Conversely, fewer modules in a gene reduce pleiotropic constraint, which should increase the rate of sequence evolution.

While Markov models for duplicate gene retention, including rates of neofunctionalization, have been developed, those for non-duplicate genes have not in the same frameworks. One reason for this is the hard assumption of selection to retain ancestral functions in these models. That assumption can be relaxed. Simulations with different sets of assumptions in these types of frameworks can be used to generate expectations of rates of orthologous vs. paralogous neofunctionalization for different types of genes. Ultimately, such models will need to be fit to data to enable characterization of actual rates with dataset-specific parameters fit.

Further, duplicating an entire pathway can lead to major biological innovation with pre-wired modules if the input and/or the output proteins diverge in function and cross-talk between duplicates dissipates through a gain of interaction specificity over time. Within networks, the role of proteins with specific functions in the overall function of the network is increasingly well understood (Kashtan et al. 2004). The network structures that generate network function can be generated by gene duplication. In fact, many of the commonly evolved modules that are recognized, like bifans and feed-forward loops, can be generated by the duplication process in a transparent manner given the functions and interactions of the pre-duplicated molecules (Kashtan et al. 2004).

## Considerations for Orthologs from this Discussion

This discussion has led us to the point where we see how novelty can be created by duplication. To the extent that paralogs can gain new functions without losing conserved ancestral ones, the same processes can happen with orthologs. One reason why this is expected to be slower/less probable involves some levels of pleiotropic constraint on the sequence as a whole, even when new functions are possible. This also will only apply to a subset of functions. If new functions involve the gain of new binding partners/loss of specificity, this might not be expected to be so hard. It should be remembered that selection for sequences that prevent binding to other potential partners constrains sequence, and therefore, removing this constraint that opens up part of sequence space becomes an event that is defined by a probability associated with the number of sequences that map to the one binding protein vs. the multiple binding proteins (Liberles et al. 2011). A general feature of long-term evolution driven by mutational pressure is that sequences constrained by not entering low fitness regions will sample functional spaces in sequence space sampled by the size of those spaces. The more designable a function is in the underlying number of sequences that can give rise to that function, the more frequently that function will evolve. This theoretical construct can give an intuitive sense of how likely a protein with one function is to evolve into a protein with a new or different one. That is, the long-term rate of functional change between proteins with any two functions will be a product of the sizes of the sequence spaces that enable those functions.

As discussed earlier, specific phylogenetic function-parameterized Markov models, both in simulation and fit to data, would be another avenue to gauge rates of orthologous neofunctionalization and to understand differences from paralogous gene evolution. At the end of this review, we are in the position of describing hypothetical models that are needed but do not exist yet.

There are many possible modeling building blocks that extend from quantitative descriptions of functions in biochemistry and systems biology. From there, several conceptual paths can lead to transitions in quantitative (and qualitative) descriptions of function that can inform a modeling framework (see McCloskey et al. (2024) for an example with metabolic pathways). Once appropriate models have been fully developed, their systematic application to data will be informative for characterizing when orthologs (and paralogs) change function. In the end, biochemistry matters.
